# Association Between Frailty and Gut Microbiota Metabolite TMAO in Older People With Cardiovascular Disease

**DOI:** 10.1093/geroni/igaa057.680

**Published:** 2020-12-16

**Authors:** Wei He, Hua Wang, Jiefu Yang

**Affiliations:** National Center of Gerontology, Beijing, China

## Abstract

The potential for the gut microbiota to affect health has particular relevance for older adults. Recent evidence suggests that microbiota-derived metabolites may modulate aging-related changes in immunity, sarcopenia, and cognitive function, all of which are elements of frailty. Trimethylamine N-oxide (TMAO) produced by the metaorganismal metabolism of choline, has been implicated in disease pathogenesis. However, relatively little geroscience research has been carried out on TMAO，and even less on other gut microbiota metabolites. The purpose of this study was to explore the relationship between frailty and circulating TMAO concentration. Data and fasting blood samples came from a prospective comprehensive geriatric assessment cohort of older adults (age≥65, n=451) with cardiovascular diseases. The frailty index based on the accumulated deficits model (48 variables) was used for evaluating the status of frailty. TMAO levels differed between groups with a significant increase for people with frailty (p＜0.001). Compared with the lowest quartile of TMAO levels, patients in the highest quartile had increased 3.07-fold risk of frailty (OR=3.07, 95%CI, 1.69-2.97). After adjusting for age, gender, BMI, history of diseases, hsCRP, LDLc, TMAO levels remained associated with frailty (OR=2.11, 95%CI, 1.01-4.38). Similarly, a cubic spline curve showed a dose-dependent relationship between the odds ratio for the risk of frailty and circulating TMAO in a linear trend (p = 0.006). This study suggests that circulating TMAO are independently associated with frailty in older adult with cardiovascular diseases. Efforts to further characterize the relationship between gut microbiota metabolite and frailty should be further pursued.

